# Supplementary material for: Systematic reviews in paediatric multiple sclerosis and Creutzfeldt-Jakob disease exemplify shortcomings in methods used to evaluate therapies in rare conditions
Source: Orphanet J Rare Dis. 2016 Feb 20;11:16. doi: 10.1186/s13023-016-0402-6 (PMC4761188; doi:10.1186/s13023-016-0402-6)
Supplement: Supplementary file 1 — Supplementary web material. Supplementary material related to this article is available online. (PDF 93 kb) [file 13023_2016_402_MOESM1_ESM.pdf]

Supplementary web material

Table S1: PRISMA checklist of 27 items (n/a: not applicable).

Section/ topic	#	Checklist item	Reported on page #
**TITLE**			
Title	1	Identify the report as a systematic review, meta-analysis, or both.	1
**ABSTRACT**			
Structured summary	2	Provide a structured summary including, as applicable: background; objectives; data sources; study eligibility criteria, participants, and interventions; study appraisal and synthesis methods; results; limitations; conclusions and implications of key findings; systematic review registration number.	2
**INTRODUCTION**			
Rationale	3	Describe the rationale for the review in the context of what is already known.	3,4,5
Objectives	4	Provide an explicit statement of questions being addressed with reference to participants, interventions, comparisons, outcomes, and study design (PICOS).	3,4,5
**METHODS**			
Protocol and registration	5	Indicate if a review protocol exists, if and where it can be accessed (e.g., Web address), and, if available, provide registration information including registration number.	n/a
Eligibility criteria	6	Specify study characteristics (e.g., PICOS, length of follow-up) and report characteristics (e.g., years considered, language, publication status) used as criteria for eligibility, giving rationale.	6
Information sources	7	Describe all information sources (e.g., databases with dates of coverage, contact with study authors to identify additional studies) in the search and date last searched.	6
Search	8	Present full electronic search strategy for at least one database, including any limits used, such that it could be repeated.	6
Study selection	9	State the process for selecting studies (i.e., screening, eligibility, included in systematic review, and, if applicable, included in the meta-analysis).	6
Data collection process	10	Describe method of data extraction from reports (e.g., piloted forms, independently, in duplicate) and any processes for obtaining and confirming data from investigators.	7
Data items	11	List and define all variables for which data were sought (e.g., PICOS, funding sources) and any assumptions and simplifications made.	7
Risk of bias in individual studies	12	Describe methods used for assessing risk of bias of individual studies (including specification of whether this was done at the study or outcome level), and how this information is to be used in any data synthesis.	7,8
Summary measures	13	State the principal summary measures (e.g., risk ratio, difference in means).	7,8
Synthesis of results	14	Describe the methods of handling data and combining results of studies, if done, including measures of consistency (e.g., I^2) for each meta-analysis.	n/a

Table S1: PRISMA checklist of 27 items (continued).

Section/ topic	#	Checklist item	Reported on page #
Risk of bias across studies	15	Specify any assessment of risk of bias that may affect the cumulative evidence (e.g., publication bias, selective reporting within studies).	n/a
Additional analyses	16	Describe methods of additional analyses (e.g., sensitivity or subgroup analyses, meta-regression), if done, indicating which were pre-specified.	n/a
**RESULTS**			
Study selection	17	Give numbers of studies screened, assessed for eligibility, and included in the review, with reasons for exclusions at each stage, ideally with a flow diagram.	8,9
Study character-istics	18	For each study, present characteristics for which data were extracted (e.g., study size, PICOS, follow-up period) and provide the citations.	9,10,11,12
Risk of bias within studies	19	Present data on risk of bias of each study and, if available, any outcome level assessment (see item 12).	12
Results of individual studies	20	For all outcomes considered (benefits or harms), present, for each study: (a) simple summary data for each intervention group (b) effect estimates and confidence intervals, ideally with a forest plot.	n/a
Synthesis of results	21	Present results of each meta-analysis done, including confidence intervals and measures of consistency.	n/a
Risk of bias across studies	22	Present results of any assessment of risk of bias across studies (see Item 15).	n/a
Additional analysis	23	Give results of additional analyses, if done (e.g., sensitivity or subgroup analyses, meta-regression [see Item 16]).	n/a
**DISCUSSION**			
Summary of evidence	24	Summarize the main findings including the strength of evidence for each main outcome; consider their relevance to key groups (e.g., healthcare providers, users, and policy makers).	13,14
Limitations	25	Discuss limitations at study and outcome level (e.g., risk of bias), and at review-level (e.g., incomplete retrieval of identified research, reporting bias).	14,15
Conclusions	26	Provide a general interpretation of the results in the context of other evidence, and implications for future research.	15,16,17
**FUNDING**			
Funding	27	Describe sources of funding for the systematic review and other support (e.g., supply of data); role of funders for the systematic review.	17

Table S2: Classification scheme of the ANN for assigning levels of evidence for therapeutic questions (see [24]).

Level of evidence	Description
Class I	RCT of the intervention of interest with masked or objective outcome assessment, in a representative population. Relevant baseline characteristics are presented and substantially equivalent among treatment groups or there is appropriate statistical adjustment for differences. The following criteria must be fulfilled:<br>(a) concealed allocation,<br>(b) primary outcome(s) clearly defined,<br>(c) exclusion/inclusion criteria clearly defined,<br>(d) adequate accounting for dropouts (with at least 80% of enrolled subjects completing the study) and crossovers with numbers sufficiently low to have minimal potential for bias.
Class II	RCT of the intervention of interest in a representative population with masked or objective outcome assessment that lacks one of the criteria (a)-(d) stated above.<br>Prospective matched cohort study with masked or objective outcome assessment in a representative population that meets the criteria (b)–(d) stated above. Relevant baseline characteristics are presented and substantially equivalent among treatment groups or there is appropriate statistical adjustment for differences.
Class III	All other controlled trials (including well-defined natural history controls or patients serving as their own controls) in a representative population, where outcome is independently assessed, or independently derived by objective outcome measurements.
Class IV	Studies not meeting class I, II, or III criteria including consensus or expert opinion.

Table S3: Summary of the characteristics on the twelve selected studies on paediatric MS.

Ref.	Study design	Primary Objectives	Endpoints	Participants	Randomisation and masking	Intervention	Control	Statistical methodology
[28]	Observ. study	Assess safety, tolerability and efficacy of interferon beta-1 a in paediatric MS patients	Safety and tolerability outcomes,<br>Relapse rate	Total analysis set: 307 patients, multiple sclerosis analysis set (with confirmed diagnosis): 298 patients	n/a	Different doses of inteferon beta-1 were given to the 307 patients	Dose-comparison controls	Descriptive statistics,<br>Poisson regression<br>Kaplan-Meier survival curves
[29]	Observ. study	Assess effects of natalizumab in patients with paediatric MS.	Relapse rate, EDSS score, frequency of patients free from clincial activity	55 patients	n/a	Treatment with natalizumab	none	Paired t-test<br>Kaplan-Meier estimates
[30]	Observ. study	Assess effects of natalizumab in patients with paediatric MS.	Relapse rate, EDSS score, number of new lesions, number of adverse events, prevalence of neutralizing antibodies against natalizumab, serum JC virus antibody status	20 patients	n/a	Treatment with natalizumab	none	Descriptive statistics, paired t-tests and Wilcoxon signed rank tests. Bonferroni correction was used to adjust for multiple testing.
[31]	Observ. study	Assess effects of immunomodulatory agents in patients with paediatric MS	Relapse rate,<br>EDSS score	130 patients with disease onset before 16 years of age	n/a	77 subjects were treated with Avonex, 36 were treated with Rebif, 3 with Betaferon and 14 with Copaxone.	Active treatment controls	Wilcoxon rank-sum test
[32]	Observ. study	Assess efficacy of beta interferon in in patients with paediatric MS	Time to first subsequent attack, severe disability occurrence	Cohort of 197 pediatric MS patients younger than 16 years of age.	n/a	Treatment with beta interferon given to 24 patients	No treatment given to 173 patients	Chi-square test , Fisher's exact test, t-test, Wilcoxon test,<br>Kaplan-Meier estimates,<br>Cox models

Table S3: Summary of the characteristics on the twelve selected studies on paediatric MS (continued).

Ref.	Study design	Primary Objectives	Endpoints	Participants	Randomisation and masking	Intervention	Control	Statistical methodology
[33]	Observ. study	Assess safety, tolerability and efficacy of interferon beta-1a	Relapse rate, EDSS score, number of adverse events	52 patients with experienced onset of symptoms of MS before age 16	n/a	Treatment with interferon beta-1a	none	Wilcoxon rank-sum test
[34]	Observ. study	Assess safety, tolerability and efficacy of interferon beta-1a	Relapse rate, EDSS score, number of adverse events	24 patients	n/a	Treatment with interferon beta-1a in different dose-escalation phases	none	Wilcoxon signed-rank test
[35]	RCT	Assess safety and efficacy of interferon beta-1a in paediatric MS patients	Relapse rate, EDSS score, new lesions, side effects	16 patients with definite relapsing-remitting MS under the age of 16	Method of randomisation is not described. No blinding.	8 patients received interferon beta-1a	8 patients received no therapy.	t-test for two independent samples
[36]	Observ. study	Assess safety, tolerability and efficacy of interferon beta-1b	Relapse rate, number of adverse events	Cohort of 43 patients with early-onset MS	n/a	Exposure to interferon beta-1b	none	Descriptive statistics
[37]	Observ. study	Assess effectiveness and safety of interferon-beta and glatiramer acetate	Relapse rate, EDSS score, number of adverse events	81 patients with, onset of the disease before age 16	n/a	51 patients were treated with interferon beta-1a once weekly, 19 with interferon beta three times weekly and 11 with glatiramer acetate.	Active treatment controls	Descriptive statistics<br>Paired t-test
[38]	Observ. study	Assess effectiveness and safety of interferon-beta and glatiramer acetate	Relapse rate, EDSS score, number of adverse events	76 patients with onset of the disease before age 16. Results evaluated for 65 subjects.	n/a	38 patients treated with interferon beta-1a once weekly (Avonex), 18 with interferon beta three times weekly (Rebif/Betaferon) and 9 with glatiramer acetate.	Active treatment controls	Descriptive statistics<br>Paired t-test
[39]	Observ. study	Assess effectiveness and safety of interferon-beta	Relapse rate, EDSS score, number of adverse events	51 patients with a disease manifestation before age 16 years.	n/a	Treatment with interferon beta-1a	None	Descriptive statistics

Table S4: Summary of the characteristics on the seven selected studies on evaluations of therapeutic interventions in CJD.

Ref.	Study design	Primary Objectives	Endpoints	Participants	Randomisation and masking	Intervention	Control	Statistical methodology
[40]	RCT	Assess efficacy of doxycycline on survival in patients with CJD	Time-to-event endpoints	121 patients (Italy: 55, France: 66) with diagnosis of definite or probable CJD	Minimisation method (Italy), simple randomisation (France); double-blind trial	Doxycycline given to 62 patients	Placebo given to 59 patients	Log-rank test, Cox model
[41]	RCT	Assess efficacy of quinacrine on survival in patients with sCJD	Time-to-event endpoints	51 patients with sCJD	Double-blind stratified randomisation; subjects were offered open-label quinacrine at month 2.	Quinacrine given to 23 patients.	Placebo given to 28 patients	Log-rank test, Cox models
[42]	Observ. study	Assess efficacy of doxycycline on survival in patients with sCJD	Time-to-event endpoints	70 patients with probable sCJD	n/a	Doxycycline given to 28 patients	42 historic patients without treatment	Median survival time, log-rank test, Cox model
[43]	Observ. study	Assess efficacy of quinacrine in patients with various prion diseases	Time-to-event endpoints	107 patients with forms of prion disease (23 in a pilot study and 84 in the main study.)	Patients were offered a choice between quinacrine, no quinacrine, or (stratfied) randomisation to immediate quinacrine or deferred quinacrine. No blinding.	Main trial: 24 chose immediate quinacrine	Main trial: 59 chose no quinacrine	Kaplan-Meier curves, log-rank tests and Cox models
[44]	Observ. study	Assess efficacy of doxycycline on survival in patients with CJD	Time-to-event endpoint	99 patients with probable CJD	n/a	Doxycycline given to 21 patients	78 patients with no treatment	Median survival time, log-rank test
[45]	Observ. study	Assess efficacy of quinacrine in patients with sCJD and vCJD.	Time-to-event endpoint, difference in Rankin score	157 patients with probable or definite sCJD or vCJD	n/a	Quinacrine given to 32 patients (2 with vCJD)	125 untreated patients with probable or definite sCJD	Mean survival time Two-sample t-test, absolute frequencies
[46]	RCT	To assess the efficacy of flupirtine on cognitive decline in patients with CJD.	Difference in ADAS-Cog score, time-to-event endpoints	28 patients with CJD	Randomised double-blind trial	Flupirtine given to 13 patients	Placebo given to 15 patients	t-test, median survival time, log-rank test
